# Connection tubing causing small bowel obstruction and colonic erosion as a rare complication after laparoscopic gastric banding: a case report

**DOI:** 10.1186/1752-1947-6-9

**Published:** 2012-01-11

**Authors:** Liza BK Tan, Jimmy BY So, Asim Shabbir

**Affiliations:** 1 National University Hospital, 5 Lower Kent Ridge Road, Singapore 119074

## Abstract

**Introduction:**

Laparoscopic adjustable gastric banding is the most frequently performed bariatric procedure for the treatment of morbid obesity and is associated with low morbidity and mortality. Complications related to obesity surgery are rare and their presentation is often non-specific. Thus, it is highly important for physicians who are practising bariatric surgery to be aware of complications described in single-case studies or series when they come across similar complications even years after the primary bariatric operation.

**Case presentation:**

We report the case of a 47-year-old Malay woman who was admitted with symptoms and signs suggesting intestinal obstruction five years after gastric band placement.

**Conclusions:**

In our patient, the band connection wire tube was the cause of both small bowel obstruction and colonic erosion. Computed axial tomography is the cornerstone of the investigation of such patients. After surgical removal of the connecting tube, our patient recovered without sequelae.

## Introduction

In this era of rising body mass indices, the need for bariatric surgery also is on the rise. Laparoscopic adjustable gastric banding (LAGB) is the most frequently performed bariatric procedure for the treatment of morbid obesity and is considered the least invasive form of bariatric surgery. LAGB, compared with other bariatric procedures, results in a shorter hospital stay, faster recovery, and cosmetically ideal scars. In addition, it is the only form of obesity surgery that is reversible. The long-term weight loss is approximately 50% of excess weight [1-4]. LAGB is associated with low morbidity and mortality. In the literature, the overall complication rate is reported to be between 9% and 13%. We report a rare late complication: a simultaneous colonic erosion and small bowel obstruction caused by a silicone connecting tube five years after an LAGB.

## Case presentation

A 47-year-old Malay woman presented with a one-day history of symptoms suggestive of intestinal obstruction, which included severe colicky abdominal pain and profuse vomiting. She had had an LAGB placement five years before. Her initial body mass index had been 59.1. Seven months after placement of the LAGB, the procedure had been complicated by a band connection wire tube leak and a port site dislocation, which necessitated a resetting of the reservoir and a trimming of the tubing. After this procedure, she was subsequently lost to follow-up. When she presented to our hospital, her body mass index was 28.2.

During a physical examination, she was noted to have abdominal distention and tenderness. The results of her laboratory test on admission showed a white blood cell count of 8.07 × 109/L. An X-ray of the abdomen revealed a loop of dilated small bowel in the upper abdomen, and air and fluid levels.

**Figure 1:**
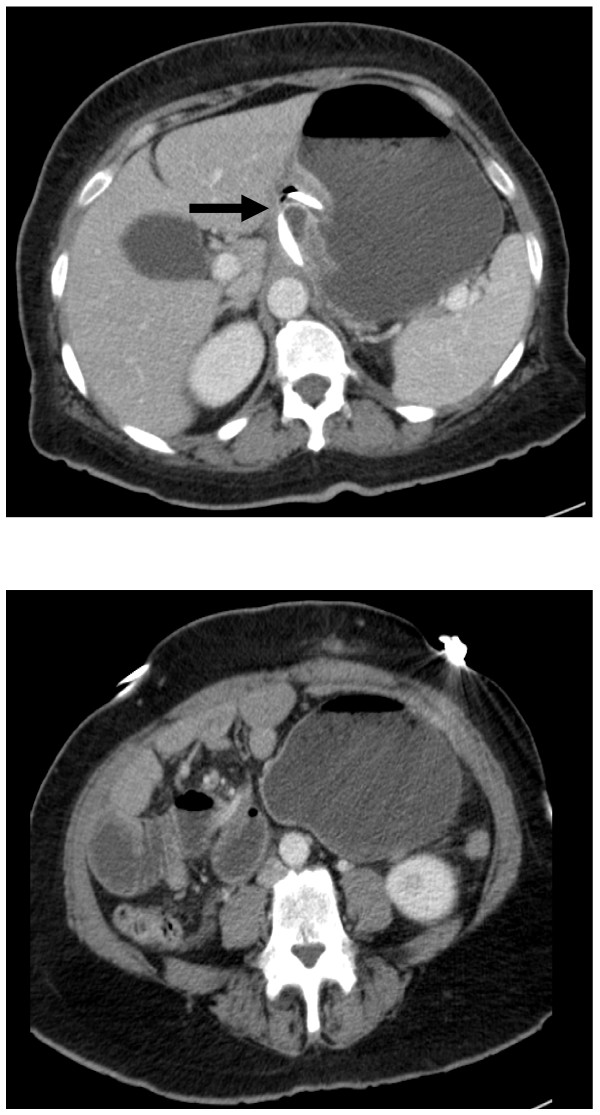
**A computed axial tomography image of the dilated stomach with a band in position**. Connection tubing can be seen wrapping around the dilated small bowel (arrow).

**Figure 2:**
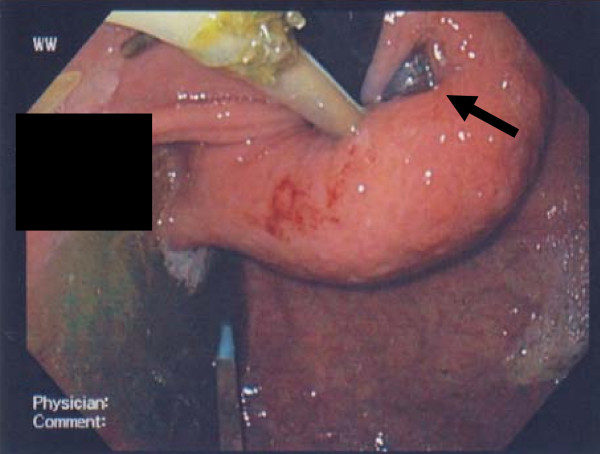
**A retroflexion view of a nasogastric tube passing through the gastroesophageal junction during a gastroscopy and a part of the band eroding though the mucosa**.

The stomach bubble was prominent. On a computed axial tomography scan (Figure 1), small bowel and stomach were noted to be dilated. The connection tubing appeared to be looping around the mesentery of the small bowel. A gastroscopy (Figure 2) demonstrated gastric band erosion of the lesser curve.

**Figure 3:**
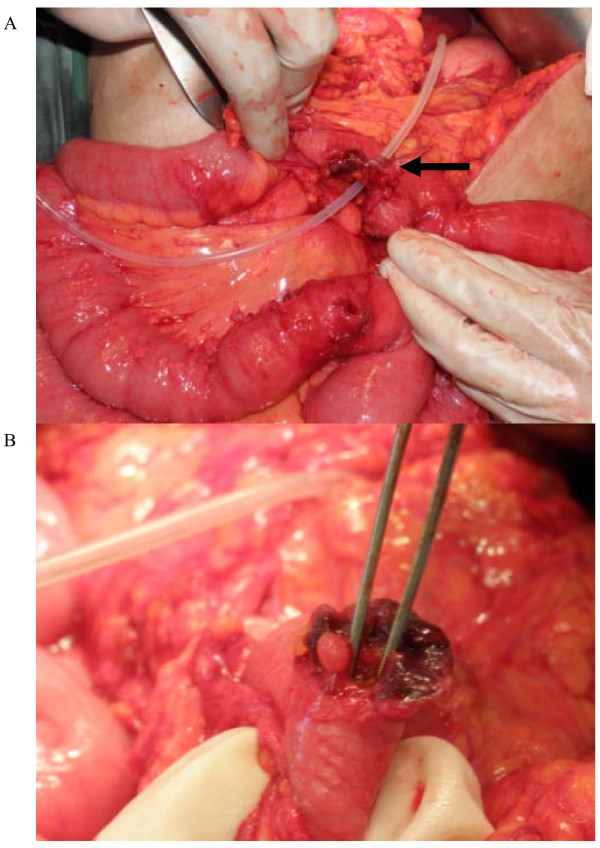
**(A) Connection tubing sandwiched between small bowel and transverse colon (arrow)**. (B) Colonic erosion.

Our patient proceeded to the operating room. During the operation, the proximal small bowel and mid-transverse colon were densely adherent to tubing. The surgeons were unable to dissect this safely, and a decision was made to convert to a full laparotomy. During the laparotomy, the adhesions were taken down sharply to reveal the tubing sandwiched between the small bowel anterior and the mid-transverse colon posteriorly (Figure 3a). It was noted, on closer inspection, that the tubing had eroded into the colon (Figure 3b). The tubing was dissected away from the mesentery and freed the small bowel, and the colonic perforation was closed with, primarily, 3.0 polydioxanone sutures in two layers. The gastric band was removed, and the lesser curve of the stomach was repaired with vicryl 2.0 sutures. A tongue of omentum was secured over the repair.

Our patient's recovery was uneventful. A gastrografin study was carried out on postoperative day five. After the absence of a contrast leak was confirmed, our patient went on a diet and was discharged on postoperative day eight. Three months after discharge, our patient had gained 15 pounds.

## Discussion

In the literature, the overall complication rate of LAGB is reported to be between 9% and 13%. Complications are generally related to the band or band connection wire tubing or the port. Band-related complications are well described in the literature.

They include the more commonly described gastric erosion (0% to 11%), pouch dilatation (5% to 17%), slippage (1% to 5%), migration (1%), and, rarely, gastric perforation (0% to 3%) [1-4]. Himpens and colleagues [5] reported that, in a 12-year period, nearly one in three patients experienced band erosions.

Although intragastric migration of adjustable bands has been widely described, colonic erosion is extremely rare and has been described only in small case series [6,7]. Similarly, case studies have described band connection wire tubing constriction around small bowel [8-12] or cecum [8] causing bowel obstruction. In one case study, a patient had severe diffuse abdominal pain in addition to port site pain as a result of traction by the band connection wire tube around the small bowel mesentery, pulling the mesentery into the right iliac fossa [9].

Interestingly, in a systemic review of band erosions after LAGB [10], a multiple regression analysis showed that the erosion rate is significantly predicted by the experience of the bariatric surgeon. Port site placement and length of band connection wire tubing have not been shown to be directly related to these complications. Intuitively, it appears that altering these factors may help in reducing these complications, but the exact method is still obscured.

## Conclusions

To the best of our knowledge, this is the first report of simultaneous gastric erosion, colonic erosion, and small bowel obstruction following gastric banding. Each complication is reported separately in case reports and case series only. These complications have not simultaneously occurred in any one patient. In this case report, we demonstrate that various combinations of complications can occur in a bariatric patient many years after the original procedure and that the cornerstone of the evaluation of bowel obstruction in these patients remains the computed axial tomography scan [13]. We have already mentioned the fact that complications of bariatric surgery remain rare and are non-specific; therefore, through the reporting of complications, we may be able to share our experience with other bariatric surgeons.

### Consent

Written informed consent was obtained from the patient for publication of this case report and any accompanying images. A copy of the written consent is available for review by the Editor-in-Chief of this journal.

## Abbreviation

LAGB: laparoscopic adjustable gastric banding.

## Competing interests

The authors declare that they have no competing interests.

## Authors' contributions

LBKT reviewed the case and prepared the manuscript. AS was the primary physician of this patient. He reviewed and treated this patient in conjunction with JBYS. Both AS and JBYS were major contributors in the writing of the manuscript. All authors read and approved the final manuscript.
